# Colour routing with single silver nanorods

**DOI:** 10.1038/s41377-019-0150-1

**Published:** 2019-04-17

**Authors:** Xiaolu Zhuo, Hang Kuen Yip, Ximin Cui, Jianfang Wang, Hai-Qing Lin

**Affiliations:** 1 Department of Physics, The Chinese University of Hong Kong, Shatin, Hong Kong SAR, China; 2 Beijing Computational Science Research Center, Beijing, 100193 China

**Keywords:** Nanoparticles, Nanophotonics and plasmonics

## Abstract

Elongated plasmonic nanoparticles have been extensively explored over the past two decades. However, in comparison with the dipolar plasmon mode that has attracted the most interest, much less attention has been paid to multipolar plasmon modes because they are usually thought to be “dark modes”, which are unable to interact with far-field light efficiently. Herein, we report on an intriguing far-field scattering phenomenon, colour routing, based on longitudinal multipolar plasmon modes supported by high-aspect-ratio single Ag nanorods. Taking advantage of the distinct far-field behaviours of the odd and even multipolar plasmon modes, we demonstrate two types of colour routing, where the incident white light can be scattered into several beams with different colours as well as different propagation directions. Because of the narrow linewidths of the longitudinal multipolar plasmon modes, there is little spectral overlap between the adjacent peaks, giving rise to outstanding colour selectivity. Our experimental results and theoretical model provide a simple yet effective picture for understanding the far-field behaviour of the longitudinal multipolar plasmon modes and the resultant colour routing phenomenon. Moreover, the outstanding colour routing capability of the high-aspect-ratio Ag nanorods enables nanoscale optical components with simple geometries for controlling the propagation of light below the diffraction limit of light.

## Optics: Colour routing on the nanoscale

Chinese scientists have developed plasmonic nanoparticles capable of controlling the direction of light of different colours, opening the door to a range of nanoscale devices for use in optical information and signal processing. Nanoscale optical components capable of wavelength routing, in which light of the same wavelength can be used repeatedly, are in high demand for constructing optical nanocircuits. However, they are challenging to fabricate due to the diffraction limit of light. Now, Jianfang Wang and colleagues from The Chinese University of Hong Kong, in collaboration with researchers from Beijing Computational Science Research Center, have created plasmonic nanoparticles from silver nanorods capable of splitting and routing light of different colours below the diffraction limit of light. The nanorods could be used for designing novel nanostructures, including optical nanoantennas, nanolasers, polarizers, and beam splitters.

## Introduction

The control of light propagation is essential for constructing optical circuits that use light or photons as the medium for carrying information and processing signals. In conventional optics, bulky optical elements such as mirrors, prisms and diffraction gratings are frequently used for modulating the wavefront of a propagating light wave, which is realized over a distance much larger than the wavelength of light. Micrometre-scale devices for wavelength routing have been demonstrated in several photonic systems, such as all-metallic gratings^1^, silicon waveguide-ring resonator coupled systems^2^ and photonic crystal waveguides^3^. Nanoscale optical components with similar functions are in strong demand for constructing optical nanocircuits, yet this is still very challenging due to the diffraction limit of light^4,5^. Localized surface plasmon resonances (LSPRs) endow plasmonic nanostructures with an outstanding ability to manipulate light at the subwavelength scale^6–9^. Rational design of plasmonic nanostructures allows for the modulation of the wavefront of propagating light in unusual ways, such as Yagi-Uda plasmonic nanoantennas for unidirectional light emission^10–12^, individual nanoparticles for light bending^13–15^, metamaterials for cloaking^16,17^, and gradient metasurfaces for light beaming^18^.

Recent progress in plasmonics has highlighted functional plasmonic nanostructures for separating light of different colours into different directions, namely, colour routing^19–24^. Colour routing allows for nanoscale light manipulation with multiple frequency channels, which is highly desired for all-optical communication devices and wavelength-encoded quantum information processing. Compared with the propagation control of single-wavelength or broadband light, colour routing is more challenging because it requires at least two types of phase modulation mechanisms with good wavelength selectivity and directionality. To date, only a few designs have been proposed and demonstrated for colour routing, such as closely spaced bimetallic dimers^19,20^, nanoantennas with circular ring gratings^21,22^, and Fano-resonance-assisted metasurfaces^23,24^. The colour routing behaviours of these nanostructures result mostly from the constructive or destructive interference of two or more dipolar LSPR modes with a specific phase retardation. Therefore, these arrangements are usually made of multiple elements with precisely designed distances and/or orientations with respect to each other, which require top-down nanofabrication techniques.

Herein, we report on two types of colour routing effects based on single high-aspect-ratio Ag nanorods, which can be easily synthesized by wet-chemistry methods. Different from prior designs, our scheme only takes advantage of the longitudinal multipolar plasmon modes, that is, high-order LSPR modes similar to the standing waves in Fabry–Pérot cavities, which are usually thought to be “dark modes” and therefore have not received much attention. The longitudinal multipolar modes at different LSPR wavelengths and with odd and even symmetry exhibit narrow plasmon bandwidths and distinct far-field scattering properties, which act together to enable colour routing with good wavelength selectivity. Two types of colour routing are experimentally observed at the single-particle level and numerically confirmed by finite-difference time-domain (FDTD) simulations. An analytical model of horizontal electric dipole arrays is further introduced to understand the far-field behaviours of the longitudinal multipolar plasmon modes and the resultant colour routing effects. Our results not only reveal the far-field angular scattering behaviours of high-aspect-ratio plasmonic nanorods but also offer the possibilities for creating optically functional nanostructures and exploring a variety of LSPR-based optical applications based on multipolar plasmon modes.

## Results

**Fig. 1:**
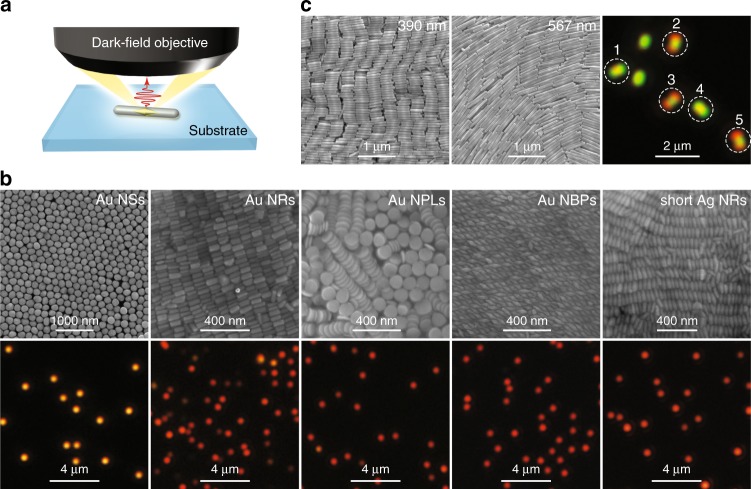
True-colour dark-field scattering images of differently shaped plasmonic nanoparticles. **a** Schematic showing the optical system for single-particle dark-field scattering imaging and spectroscopy. **b** SEM (upper row) and dark-field (lower row) images of the Au nanospheres (Au NSs, average diameter: 194 ± 12 nm), Au nanorods (Au NRs, average length/diameter: 100 ± 10 nm/45 ± 6 nm), Au nanoplates (Au NPLs, average diameter/thickness: 112 ± 4 nm/22 ± 1 nm), Au nanobipyramids (Au NBPs, average length/waist diameter: 99 ± 4 nm/33 ± 2 nm), and short Ag nanorods (Ag NRs, average length/diameter: 110 ± 5 nm/38 ± 3 nm). **c** SEM (left and middle) and dark-field scattering (right) images of two Ag nanorod samples with average lengths/diameters of 390 ± 46 nm/64 ± 3 nm and 567 ± 85 nm/66 ± 3 nm, respectively. The scattering image was taken from a mixture of the 390-nm and 567-nm Ag nanorod samples. The nanoparticle samples were deposited on highly doped Si substrates for SEM imaging and on cover glass for the scattering measurements

The colour routing effects came to our attention through single-particle dark-field scattering measurements (Fig. 1a) on individual plasmonic nanoparticles of different shapes. Cover glass (SiO_2_) was chosen as the substrate because of its low refractive index (~1.45), which results in the smallest substrate effect on the LSPRs and the resultant far-field behaviours^25,26^. Five types of commonly used plasmonic nanoparticles, including Au nanospheres^27^, Au nanorods^28^, Au nanoplates^29^, Au nanobipyramids and Ag nanorods^30–32^, were synthesized by seed-mediated growth methods and randomly deposited on cover glass at low surface number densities (0.1–0.4 μm^–2^) for the dark-field scattering measurements. Figure 1b, c shows scanning electron microscopy (SEM) images of the nanoparticles, together with the real-colour dark-field scattering images of individual nanoparticles at the air–glass interface. For the samples shown in Fig. 1b, the single-particle dark-field scattering images appear as solid bright spots, regardless of the geometry and composition of the nanoparticles. The solid bright spots with red colour are due to the excitation of dipolar plasmon modes at relatively long wavelengths in the visible region (Supplementary Fig. S1a). Although we chose only one typical size as an example for each type of nanoparticle, we believe that this type of solid bright spot pattern is a universal phenomenon for most plasmonic nanoparticles supported on low-index substrates. Similar patterns have also been observed in many other plasmonic nanoparticles of different sizes and shapes^33^.

The two Ag nanorod samples shown in Fig. 1c were synthesized through Au nanobipyramid-directed Ag overgrowth using different amounts of the silver precursor to tailor the average rod length^30–32^. When the two samples are dispersed in water, their transverse dipolar plasmon peaks are both at ~400 nm, and their longitudinal dipolar plasmon peaks are both in the near-infrared region exceeding 1200 nm (Supplementary Fig. S1b), with several lower-intensity peaks originating from longitudinal multipolar plasmon modes of different orders^30,34,35^. The two samples were mixed in water and then deposited on cover glass for dark-field scattering imaging. Surprisingly, we observed intriguing single-particle dark-field scattering images with double red spots (the images labelled 2, 3 and 5 in Fig. 1c) and double green spots (the images labelled 1 and 4 in Fig. 1c). To confirm that these intriguing images result from individual Ag nanorods, we repeated the dark-field scattering measurements using Si wafers with a 300-nm-thick thermal oxide layer as substrates and carried out SEM imaging by the pattern-matching method^30,34^. The Ag nanorods with an average length of 303 ± 5 nm were found to show double red spots, and those with an average length of 559 ± 27 nm were found to give double green spots (Supplementary Fig. S2). These images are slightly different from those shown in Fig. 1c because of the thin film interference resulting from the thermal oxide layer (Supplementary Fig. S3). Therefore, the oxide-coated Si wafers were only used for correlating the orientation observed under SEM and the dark-field image of each Ag nanorod. The length axis of the Ag nanorod was found to be along the elongated direction of the dark-field scattering image. In other words, the orientations of the individual plasmonic nanorods can be easily identified by conventional dark-field scattering imaging, without the use of the additional techniques of defocusing^36,37^, polarization analysis^38–40^, surface-enhance fluorescence^41^, or high-order laser modes^42^.

**Fig. 2:**
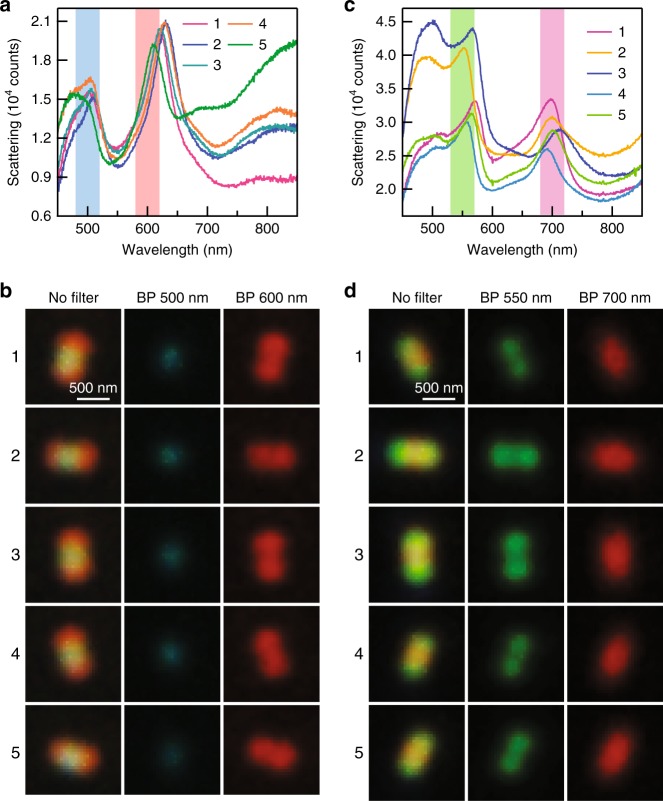
Representative dark-field scattering phenomena of the individual Ag nanorods. **a** Dark-field scattering spectra measured from five Ag nanorods showing the red-blue-red patterns. The two vertical bars indicate the bandpass colour filters. The plasmon peaks at ~500 nm arise from the longitudinal octupolar plasmon mode (*N* = 3). The plasmon peaks at ~650 nm arise from the longitudinal quadrupolar plasmon mode (*N* = 2). **b** Dark-field scattering images of the five Ag nanorods shown in **a**. The images were recorded in the absence of a colour filter (left column), with the 500-nm bandpass filter (middle column) and the 600-nm bandpass filter (right column) inserted in front of the colour camera. **c** Dark-field scattering spectra measured from five Ag nanorods showing the green-red-green patterns. The two vertical bars indicate the bandpass colour filters. The plasmon peaks at ~550 nm arise from the longitudinal hexadecapolar plasmon mode (*N* = 4). The plasmon peaks at ~700 nm arise from the longitudinal octupolar plasmon mode (*N* = 3). **d** Dark-field scattering images of the five Ag nanorods shown in (**c**). The images were measured in the absence of a colour filter (left column), with the 550-nm bandpass filter (middle column) and the 700-nm bandpass filter (right column) inserted in front of the colour camera. The Ag nanorod samples were deposited on cover glass for the optical measurements

We are particularly interested in the dark-field images with double red/green spots because this phenomenon has never been reported so far, neither from plasmonic nanoparticles nor any other nanoparticles. We therefore selected the individual Ag nanorods showing such intriguing patterns and measured their single-particle scattering spectra. Figure 2a, b shows the dark-field scattering spectra and images of five representative Ag nanorods that give the double red spot patterns. All of them simultaneously support two plasmon peaks at ~500 nm and ~650 nm in the visible region, corresponding to the scattered light with blue and red colours, respectively. For a better visualization, two bandpass colour filters were used to separate the blue and red colours from the original dark-field images (Supplementary Fig. S4), giving rise to a blue spot at the centre and two red spots at the two ends of each image. In the absence of any colour filter, the white spot at the centre of each coloured pattern is caused by a superposition of the red and blue colours following the red-green-blue (RGB) colour model. Similarly, we also found that each of the dark-field images with double green spots consists of two green spots at the ends and a red spot at the centre. Five representative examples are provided in Fig. 2c, d for the scattering spectra and images, respectively. According to our previous study on the dependence of the multipolar plasmon wavelengths on the nanorod length^30^, the two plasmon peaks shown in Fig. 2a are associated with the longitudinal octupolar (*N* = 3) and quadrupolar (*N* = 2) plasmon modes, while those shown in Fig. 2c correspond to the hexadecapolar (*N* = 4) and octupolar (*N* = 3) plasmon modes, respectively. The distinct far-field behaviours of these longitudinal multipolar plasmon modes give rise to the red-blue-red and green-red-green patterns, as will be further revealed by simulations below. Multipolar plasmon modes are usually thought to be “dark” and inaccessible by far-field light^43,44^. However, since the lengths of these Ag nanorods are on the order of visible light wavelengths, the retardation effect is important in this case so that the longitudinal multipolar plasmon modes can be excited and detected by far-field imaging and spectroscopy^45^. A prior study has demonstrated that, compared with the longitudinal dipolar plasmon mode, these longitudinal multipolar plasmon modes exhibit weaker radiative damping and therefore possess narrower linewidths in the scattering spectra^46^. Benefiting from the narrow linewidths, the adjacent peaks have little spectral overlap, resulting in the sharp colour contrast in the dark-field images.

**Fig. 3:**
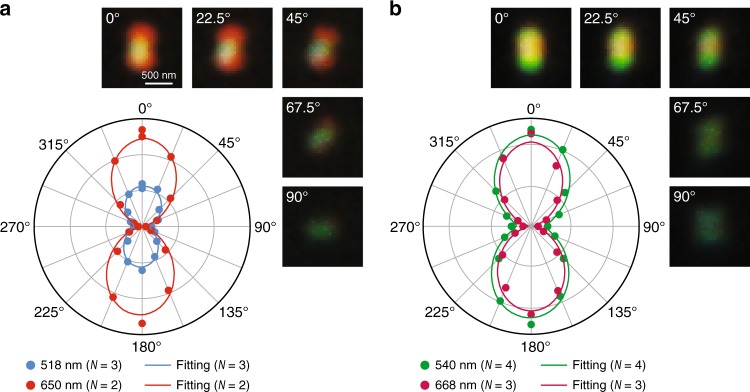
Emission polarization features. **a** Dark-field scattering images and polar plots of the scattering peak intensities measured from a single Ag nanorod showing the red-blue-red pattern. The polar plots of the scattering intensities were extracted at 518 nm for the *N* = 3 mode and at 650 nm for the *N* = 2 mode as functions of the analyser polarization direction. **b** Dark-field scattering images and polar plots of the scattering peak intensities measured from a single Ag nanorod showing the green-red-green pattern. The polar plots of the scattering intensities were extracted at 540 nm for the *N* = 4 mode and at 668 nm for the *N* = 3 mode as functions of the analyser polarization direction. The analyser polarization direction was varied from 0° to 360° at a step of 22.5°. Only the scattering images in the first quadrant are shown. The polar plots were fitted by sine squared functions. The coefficients of determination (*R*^2^) for the fitting are all above 0.95. The Ag nanorods were supported on cover glass

For both cases, polarization measurements were further performed by inserting a polarization analyser in front of the detection camera. The recorded scattering spectra, as well as the dark-field images, vary with the analyser polarization direction (Fig. 3 and Supplementary Fig. S5). The intensities of the plasmon peaks and the dark-field images are maximized when the analyser polarization axis is aligned parallel to the length axis of the Ag nanorod, while they fade out when the analyser polarization axis is perpendicular to the length axis. Figure 3a, b shows polar plots of the peak intensities for the two cases. Both plots can be well fitted with sine squared functions, indicating that the scattered light is oriented parallel to the nanorod length axis. The polarization-dependent behaviour clearly confirms that the red-blue-red and green-red-green images both originate from the longitudinal plasmon modes. The transverse plasmon mode does not contribute to the dark-field scattering images because its wavelength is close to the edge of the visible region and beyond the detection limit of our colour camera.

**Fig. 4:**
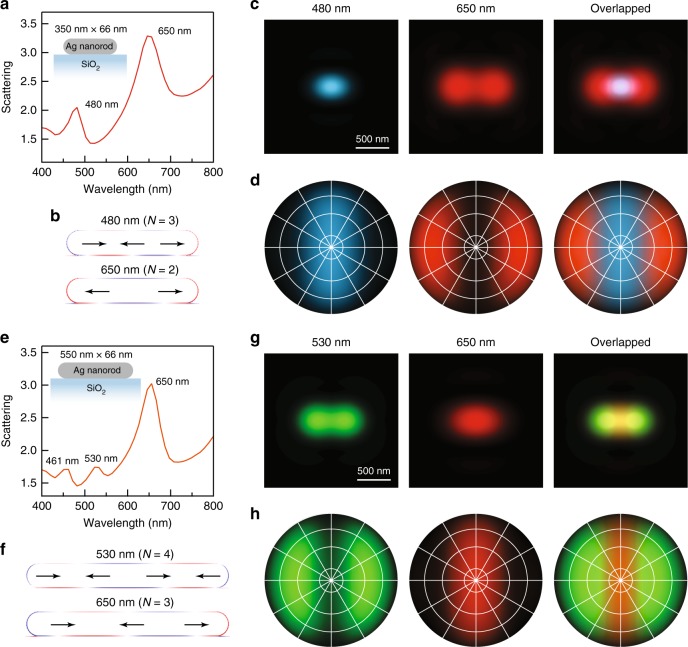
FDTD simulation results. **a** Simulated scattering spectrum of a SiO_2_-supported Ag nanorod with a length of 350 nm and a diameter of 66 nm. **b**–**d** Simulated charge distribution contours, real-space images and back focal plane images of the 480-nm and 650-nm plasmon peaks shown in (**a**), respectively. **e** Simulated scattering spectrum of a SiO_2_-supported Ag nanorod with a length of 550 nm and a diameter of 66 nm. **f**–**h** Simulated charge distribution contours, real-space images and back focal plane images of the 530-nm and 650-nm peaks shown in (**e**), respectively. The overlapped real-space and back focal plane images were generated by combining images with different colours. The number above each pattern is the corresponding plasmon wavelength. The central point and the four circles from the inside to outside of the back focal plane images represent 0°, 10°, 30°, 60° and 90° relative to the normal direction of the substrate from the top view. The horizontal axis is along the length direction of the Ag nanorod. The charge oscillations of the plasmon bands can be simplified as a few horizontally aligned electric dipoles, as illustrated by the black arrows in the charge distribution contours

To reveal the physical origin of the observed red-blue-red and green-red-green patterns, FDTD simulations were performed to analyse the near-field and far-field properties of the plasmon modes supported by the individual Ag nanorods. Both nanorods were modelled as cylinders with two hemispherical ends. A spacing of 1 nm was inserted between the nanorod and the substrate due to the presence of the cetyltrimethylammonium bromide (CTAB) capping layer^47^. To simulate the Ag nanorods showing the red-blue-red patterns, we considered a silver nanorod with a length of 350 nm and a diameter of 66 nm. The simulated scattering spectrum shows two plasmon peaks at 480 nm and 650 nm (Fig. 4a), which can be identified from their charge distribution contours (Fig. 4b) to be the *N* = 3 and *N* = 2 plasmon modes, respectively. We further simulated the far-field real-space image by combining the contributions of the two plasmon modes and plotting the results with the corresponding colours (Fig. 4c). As a result, a red-blue-red real-space image was obtained. The image is very similar to our experimental results, which confirms that such an intriguing pattern is caused by the different far-field behaviours of the *N* = 3 and *N* = 2 plasmon modes. The different far-field behaviours can be seen more clearly from the simulated back focal plane Fourier images, which are angular intensity distributions of the scattered light with specific colours. The results shown in Fig. 4d imply that blue light is scattered along the waist direction of the Ag nanorod and that the red light is scattered obliquely towards the two ends of the nanorod. In other words, this is a colour routing effect for separating blue and red light into different spatial directions. We have made great effort on back focal plane imaging measurements to verify our results. However, it is very challenging to perform back focal plane imaging at the single-particle level in our dark-field scattering system due to the weak signals and small signal-to-noise ratios. Nevertheless, we believe that the real-space images obtained in our experiments can provide circumstantial evidence for the colour routing effect.

For the Ag nanorods showing the green-red-green patterns, we considered a silver nanorod with a length of 550 nm and a diameter of 66 nm in the simulation (Fig. 4e). The difference in the relative peak intensities between the simulated and experimental scattering spectra is believed to be caused by the different light collection efficiencies of the simulation monitor and the dark-field objective used. As the length of the Ag nanorod is increased from 350 nm to 550 nm, the *N* = 3 and *N* = 2 plasmon peaks redshift (Fig. 4f), while additional peaks of higher orders appear on the short-wavelength side. As a result, the *N* = 3 plasmon mode shifts to 650 nm; therefore, red light is scattered along the waist direction of the nanorod in this case (Fig. 4g, h). Green light, associating with the *N* = 4 plasmon mode, is scattered towards the two ends of the Ag nanorod. Taken together, the green-red-green patterns observed in our experiments can be attributed to the distinct far-field behaviours of the *N* = 4 and *N* = 3 plasmon modes. This is another type of colour routing that relies solely on the longitudinal multipolar plasmon modes. In addition, there is an *N* = 5 plasmon peak, corresponding to blue light, both in the simulated spectrum and the experimental scattering spectra. The simulated real-space and back focal plane images of the single Ag nanorod, either taking the *N* = 5 plasmon mode into consideration or not, are displayed in Fig. 4h and Supplementary Fig. S6 for comparison. In general, the *N* = 5 plasmon mode does not result in any significant difference in the final image except a slight colour change due to its weak intensity. For the purpose of simplicity, we will only focus on the *N* = 4 and *N* = 3 plasmon modes for the green-red-green patterns in the following discussion.

**Fig. 5:**
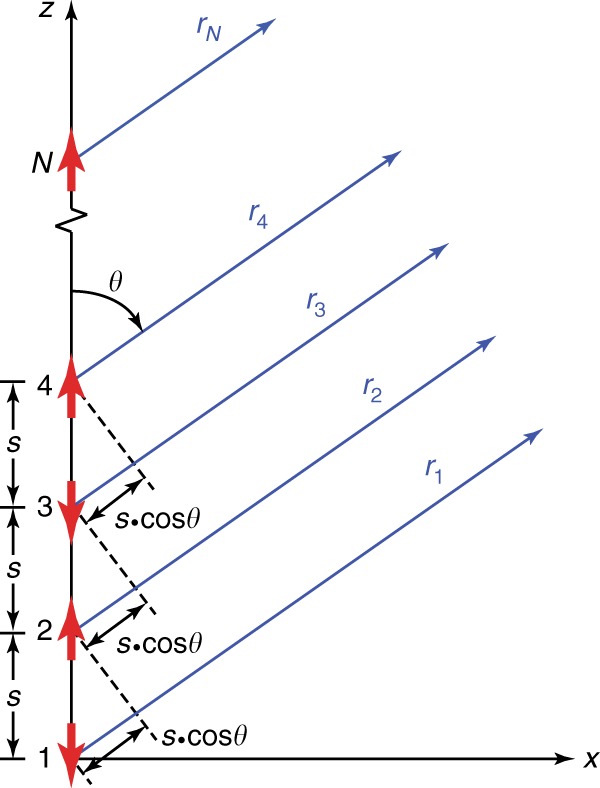
Schematic of the electric dipole array model. There are *N* elements in the array. The red arrows denote the individual electric dipoles in an out-of-phase alignment with a uniform spacing *s*. The blue arrows refer to the distances from the dipoles to a detection point far away from the array

As illustrated in the charge distribution contours in Fig. 4b, f, the charge oscillations of the longitudinal multipolar plasmon modes can be simplified to electric dipole arrays with *N* elements. To further understand their far-field behaviours, a theoretical model of *N*-element electric dipole arrays is proposed to provide a simple physical picture of the colour routing effects. For the purpose of simplicity, we assume that the *N* electric dipoles are aligned parallel to the length axis of the Ag nanorod with a uniform spacing *s* and a unit amplitude for each dipole, as illustrated in Fig. 5. The electric dipole in the perpendicular direction, corresponding to the transverse plasmon mode, is not considered in our model because of its limited contribution to the colour routing effect in our experiments. Assuming that there is no near-field coupling between the adjacent dipoles, the total field radiation from the electric dipole array to the far-field can be considered as the sum of the radiation from each electric dipole and expressed as^48^

1 $${\mathbf{E}}_{{\mathrm{total}}} 	= {\mathbf{E}}_1 + {\mathbf{E}}_2 + {\mathbf{E}}_3 + \cdots + {\mathbf{E}}_{\mathrm{N}}\\ 	= {\mathbf{E}}_{{\mathrm{single}}}\left[ 1 + e^{j\left( {ks{\mathrm{cos}}\theta + \beta } \right)} + e^{j2\left( {ks{\mathrm{cos}}\theta + \beta } \right)} + \cdots\right. \\ 	{\hskip 10pt} + \left. e^{j\left( {N - 1} \right)\left( {ks{\mathrm{cos}}\theta + \beta } \right)} \right]\\ 	= {\mathbf{E}}_{{\mathrm{sing}}le}\mathop {\sum}\nolimits_{n = 1}^N {e^{j\left( {n - 1} \right)\left( {ks{\mathrm{cos}}\theta + \beta } \right)}}$$

where ${\mathbf{E}}_{{\mathrm{single}}} = - \frac{{\omega ^2\mu _0p_0}}{{4\pi }}{\mathrm{sin}}\theta \frac{{e^{j\omega \left( {r/c - t} \right)}}}{r}\hat \theta$ is the radiated far-field from a single electric dipole that harmonically oscillates in time. In the two expressions of the far-field radiation above, *ω* and *p*_0_ are the angular frequency and dipole moment of the electric dipole, respectively, *μ*_0_ and *c* are the permeability of free space and the speed of light in vacuum, *r* (*r* >> *s*) is the distance from the dipole, *k* = *ω*/*c* is the wavenumber, $\hat \theta$ is the unit vector of the polar angle, *β* is the difference in the excitation phase between the adjacent dipoles, and *t* and *j* represent time and the imaginary unit. $\mathop {\sum}\nolimits_{n = 1}^N {e^{j\left( {n - 1} \right)\left( {ks{\mathrm{cos}}\theta + \beta } \right)}}$ can be defined as an array factor (AF), which is determined by how the electric dipoles are aligned. According to the charge distribution contours obtained from the FDTD simulations, *β* = *π* due to the antiparallel alignment. The AF therefore becomes

2 $$\mathrm{AF} = {\sum}_{n = 1}^N {e^{j\left( {n - 1} \right)\left( {ks{\mathrm{cos}}\theta + \pi } \right)}} = \frac{{e^{jN\left( {ks{\mathrm{cos}}\theta + \pi } \right)} - 1}}{{e^{j\left( {ks{\mathrm{cos}}\theta + \pi } \right)} - 1}}\\ =	 \, e^{j\left[ {\left( {N - 1} \right)/2} \right]\left( {ks{\mathrm{cos}}\theta + \pi } \right)}\frac{{e^{j\left( {N/2} \right)\left( {ks{\mathrm{cos}}\theta + \pi } \right)} - e^{ - j\left( {N/2} \right)\left( {ks{\mathrm{cos}}\theta + \pi } \right)}}}{{e^{j\left( {1/2} \right)\left( {ks{\mathrm{cos}}\theta + \pi } \right)} - e^{ - j\left( {1/2} \right)\left( {ks{\mathrm{cos}}\theta + \pi } \right)}}}\\ =	 \, e^{j\left[ {\left( {N - 1} \right)/2} \right]\left( {ks{\mathrm{cos}}\theta + \pi } \right)}\frac{{{\mathrm{sin}}\left[ {\left( {N/2} \right)\left( {ks{\mathrm{cos}}\theta + \pi } \right)} \right]}}{{{\mathrm{sin}}\left[ {\left( {1/2} \right)\left( {ks{\mathrm{cos}}\theta + \pi } \right)} \right]}}$$

The angular radiation field can then be expressed as

3 $${\mathbf{E}}_{\rm{total}} =	 {\mathbf{E}}_{{\rm{single}}} \times {\mathrm{AF}} \\ =	 - \frac{{\omega ^2\mu _0p_0}}{{4\pi }}{\mathrm{sin}}\theta \frac{{e^{j\omega \left( {r/c - t} \right)}}}{r}\\ 	e^{j\left[ {\left( {N - 1} \right)/2} \right]\left( {ks{\mathrm{cos}}\theta + \pi } \right)}\frac{{{\mathrm{sin}}\left[ {\left( {N/2} \right)\left( {ks{\mathrm{cos}}\theta + \pi } \right)} \right]}}{{{\mathrm{sin}}\left[ {\left( {1/2} \right)\left( {ks{\mathrm{cos}}\theta + \pi } \right)} \right]}}\hat \theta $$

As a result, the angular radiation power can be described by

4 $$\left| {{\mathbf{E}}_{{\rm{total}}}} \right|^2 \propto {\mathrm{sin}}^2\theta \frac{{{\mathrm{sin}}^2\left[ {\left( {N/2} \right)\left( {ks{\mathrm{cos}}\theta + \pi } \right)} \right]}}{{{\mathrm{sin}}^2\left[ {\left( {1/2} \right)\left( {ks{\mathrm{cos}}\theta + \pi } \right)} \right]}}$$

**Fig. 6:**
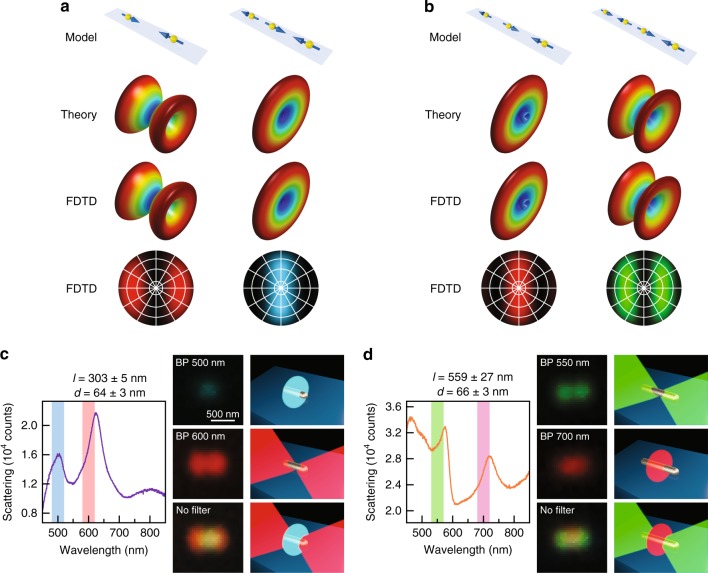
Physical picture for the colour routing effects. **a**, **b** Schematics of the theoretical models (first row), theoretically calculated 3D far-field radiation patterns (second row), FDTD-simulated 3D far-field radiation patterns (third row) and FDTD-simulated back focal plane images (fourth row) of the horizontally aligned electric dipole arrays based on the charge distribution contours shown in Fig. 4b, f, respectively. **c** Understanding of the colour routing effect for the red-blue-red patterns. The left side is the experimental scattering spectrum of a typical nanorod showing the red-blue-red pattern, with the two vertical bars indicating the bandpass colour filters. The middle column shows the experimental scattering images acquired with the 500-nm bandpass filter (first row), with the 600-nm bandpass filter (second row) and in the absence of a colour filter (third row) in front of the camera, together with the corresponding schematics for understanding the colour routing effect (right column). **d** Understanding of the colour routing effect for the green-red-green patterns. The left side is the experimental scattering spectrum of a typical nanorod showing the green-red-green pattern, with the two vertical bars indicating the bandpass colour filters. The middle column shows the scattering images acquired with the 550-nm bandpass filter (first row), with the 700-nm bandpass filter (second row) and in the absence of a colour filter (third row) in front of the camera, together with the corresponding schematics for understanding the colour routing effect (right column)

When *N* = 1, Eq. (4) becomes $\left| {{\mathbf{E}}_{{\mathrm{total}}}} \right|^2 = | {{\mathbf{E}}_{{\mathrm{single}}}} |^2 \propto {\mathrm{sin}}^2\theta$, corresponding to the dipolar plasmon resonance with a doughnut-shaped three-dimensional (3D) far-field pattern, which can be used for understanding the far-field scattering images of the plasmonic nanoparticles shown in Fig. 1b. For example, the low-aspect-ratio Au nanorods can be treated as single electric dipoles oriented parallel to the substrate plane, leading to the dark-field scattering images detected by the objective as solid bright spots. For the electric dipole arrays with multiple elements (*N* > 1), the 3D far-field patterns were calculated according to the dipole number *N*, the dipole resonance wavelength (*λ* = 2*π*/*k*) and the spacing *s* that were determined from the FDTD simulation results. Taking the 350-nm-long Ag nanorod as an example, the quadrupolar mode corresponds to an electric dipole array with *N* = 2, *λ* = 650 nm and *s* = 180 nm, and the octupolar mode corresponds to an electric dipole array with *N* = 3, *λ* = 480 nm and *s* = 120 nm (Fig. 6a). The plasmon modes of the 550-nm-long Ag nanorod can be modelled in the same manner (Fig. 6b). We note that a strong approximation was used in the derivation of Eq. (4), that is, the assumption that there is no near-field coupling between the adjacent dipoles. FDTD simulations were therefore further performed by including the inter-dipole near-field coupling to calculate the 3D far-field radiations of the electric dipole arrays and to compare the results with the results calculated according to the theoretical model. The horizontal electric dipole arrays considered in the FDTD simulations had the same geometric parameters and resonance wavelengths as those in the theoretical model. As shown in Fig. 6a, b, the FDTD simulation and theoretical results coincide well with each other, indicating that the inter-dipole near-field interaction is negligible. The arrays with odd and even numbers of electric dipoles give two different types of 3D far-field patterns, one with a disk-like main lobe at the centre and another with two ear-like sideway lobes. In both cases, there is no energy radiated along the longitudinal axis of the array, which is similar to the case of a single electric dipole. A key difference between the odd and even cases is the superposition of the electromagnetic field at the central plane perpendicular to the longitudinal axis. Qualitatively, for the arrays with an even number of elements, there are electric dipole pairs with opposite orientations located on the two sides of the central plane symmetrically, leading to a complete field cancellation and therefore no power radiation along the central plane. As a result, the power from the electric dipoles can only be radiated obliquely into the far field, forming the ear-like lobes. For the arrays with an odd number of elements, the electromagnetic waves from the dipoles cannot fully cancel with each other, which results in power radiation along the central plane. When the inter-dipole spacing is at a subwavelength scale, the odd arrays possess 3D far-field patterns similar to the pattern of a single electric dipole due to their overall nonzero net dipole moments. When viewed from above, the corresponding back focal plane images of the electric dipole arrays can be obtained. The results are plotted with specific colours determined by the resonance wavelengths of the electric dipoles in the arrays. A comparison of these results with the simulated back focal plane images of the single Ag nanorods shown in Fig. 4 confirms that the far-field behaviours of the longitudinal multipolar plasmon modes and the colour routing effects can be effectively described by the horizontally aligned electric dipole array model.

## Discussion

All of the 3D far-field patterns in Fig. 6 are rotationally symmetric about the length axis of the nanorod because of the geometrical symmetry of the electric dipole arrays. The arrays with *N* = 2 and *N* = 4 radiate power towards the two ends of the electric dipole array. In contrast, for the arrays of *N* = 3 in both cases, the radiated power is highly concentrated along the waist directions perpendicular to the electric dipole arrays. Similar far-field patterns have been reported in several previous theoretical works on far-field scattering/emission of plasmonic nanorods using classical electrodynamic simulations or analytical models of a point-source excitation^49,50^. Our model provides a much simpler analytical solution to this problem. It is worth noting that these 3D far-field patterns not only reflect how the light energy is radiated from the near field to the far field but also indicate the angle-dependent excitation efficiencies of the different multipolar modes, that is, the energy coupling efficiencies from the far field to the near field. Therefore, these results are also useful for understanding the selective excitation of longitudinal multipolar plasmon modes^51^, different collection efficiencies of the scattered light from odd and even plasmon modes using dark-field objectives^52^, and the excitation and emission of nanoscale emitters coupled to longitudinal multipolar plasmon modes^53^. As an example, we performed FDTD simulations on an electric dipole emitter positioned on a high-aspect-ratio Ag nanorod (Supplementary Fig. S7). When the emitter is efficiently coupled to the Ag nanorod, its radiation can be largely enhanced due to the Purcell effect. The corresponding 3D emission patterns are similar to those shown in Fig. 6a, b, suggesting a colour-dependent nanoscale source beyond a simple Hertz dipole source. These features are highly desired for various on-chip nanophotonic devices, such as nanolasers, optical demultiplexers and quantum communication components. In addition, the colour routing effect is expected to hold for collective nanorods as long as the nanorods are separated from each other at a distance large enough for the prohibition of plasmon coupling and are aligned parallel to each other, as has been demonstrated in other colour routing schemes^19,20^. On the other hand, we admit that the directionality of our single nanorod system is moderate compared with other examples with more complicated designs, such as Yagi-Uda nanoantennas^10–12^ and bimetallic dimers^19,20^. More effort will be required for the development of a real integrated optical wavelength router with high-aspect-ratio Ag nanorods.

Furthermore, it is worth pointing out that our dark-field measurements were carried out by placing the Ag nanorods at the air–glass interface. As a result, the 3D far-field patterns should have asymmetric lobes that preferentially penetrate into the substrate due to the asymmetric dielectric surrounding environment^53^. Nevertheless, we believe our theoretical model can provide a simple physical picture for understanding the far-field behaviours of longitudinal multipolar plasmon modes and the resultant colour routing effects, as illustrated in Fig. 6c, d. However, the colour routing effects would be strongly suppressed if the Ag nanorods were deposited on high-refractive-index substrates, such as SiC, Si and Ge, whose refractive indexes are approximately 2.7, 3.6 and 4.3, respectively, in the visible region. The strong substrate effect can induce another LSPR mode that is aligned along the transverse direction of the Ag nanorods. This mode is associated with charge oscillations perpendicular to the substrate surface, as have been carefully investigated in our recent work^54^.

In summary, we have investigated two types of colour routing effects enabled by longitudinal multipolar plasmon resonances in high-aspect-ratio Ag nanorods. The unconventional dark-field images of individual Ag nanorods, which show red-blue-red or green-red-green patterns, are revealed to be a result of the different far-field behaviours of longitudinal multipolar plasmon resonances with odd and even symmetries. FDTD simulations show that the scattered light of different colours can be sorted into different directions around the nanorod. Compared with the colour routing effects reported in prior works, the colour routing phenomena observed from high-aspect-ratio Ag nanorods rely solely on the multipolar plasmon modes, which possess narrow spectral linewidths and therefore good colour selectivity. Moreover, we have proposed an analytical model of horizontally aligned electric dipole arrays to provide a simple physical picture for understanding the colour routing effects. Our results also suggest that the multipolar plasmon modes are not necessarily “dark modes” as they were thought to be. They can also be employed for designing optical nanoantennas, nanolasers, polarizers, beam splitters, multiplexing modulators, and other nanoscale optical components. We can foresee that in addition to the colour routing effects demonstrated in our work, a variety of novel and interesting nanostructures for light manipulation can be developed based on various multipolar plasmon modes.

## Materials and methods

### Synthesis of the plasmonic nanoparticles

The Ag nanorod samples were synthesized by Au nanobipyramid-directed growth. The Au nanophere, nanorod, nanoplate and nanobipyramid samples were prepared by seed-mediated growth.

### Optical characterization

Single-particle dark-field scattering spectroscopy and imaging were performed with an upright optical microscope (Olympus, BX53M) integrated with a quartz–tungsten–halogen lamp (100 W), a digital colour camera with a resolution of 1200 × 1600 pixels (Olympus, DP73), and a monochromator (Princeton Instruments, SP2300i, cooled to −70 °C). A 100× dark-field objective with a numerical aperture of 0.9 was used for both the excitation of the individual nanoparticles with white light and the collection of the scattered light. For the emission polarization characterization, a linear polarizer (U-AN360, Olympus) was placed in front of the camera. A pattern-matching method was applied to correlate the optical images from single-particle dark-field scattering characterization with the geometrical structure of the individual nanoparticles from SEM imaging.

### Electrodynamic simulations

All of the electrodynamic simulations were performed using FDTD Solutions 8.7 developed by Lumerical Inc. The dielectric function of Ag was calculated by fitting the experimental data of Palik. The refractive index of the substrate was set to 1.45 for cover glass and that of the surrounding medium was set to 1.00 for air. Directly above the Ag nanorod in the simulation model, a two-dimensional (2D) power monitor was employed for recording the near-field information of the scattered light. The data obtained from the monitor were post-processed by near- to far-field projection. For the calculation of the back focal plane images, the data obtained from the monitor were decomposed into a series of plane waves, which propagate along different directions. In the subsequent calculation of the real-space images, plane waves with angles outside of the numerical aperture were discarded, and the remaining plane waves were re-focused onto an image plane. Both of the above calculations were carried out using the analysis scripts provided by Lumerical. The 2D and 3D far-field patterns of the electric dipole arrays were simulated using single-frequency point sources aligned in a line with alternating orientations and a certain spacing from each other.

## Supplementary information

Supplementary Information-Color routing on single silver nanorods
