# Supplementary material for: Colour routing with single silver nanorods
Source: Light Sci Appl. 2019 Apr 17;8:39. doi: 10.1038/s41377-019-0150-1 (PMC6467987; doi:10.1038/s41377-019-0150-1)
Supplement: Supplementary file 1 — Supplementary Information-Color routing on single silver nanorods [file 41377_2019_150_MOESM1_ESM.docx]

Supplementary Information

Colour routing with single silver nanorods

Xiaolu Zhuo^1^, Hang Kuen Yip^1^, Ximin Cui^1^, Jianfang Wang^1^* and Hai-Qing Lin^2^

^1^Department of Physics, The Chinese University of Hong Kong, Shatin, Hong Kong SAR, China; ^2^Beijing Computational Science Research Center, Beijing 100193, China

*Corresponding author, e-mail: jfwang@phy.cuhk.edu.hk


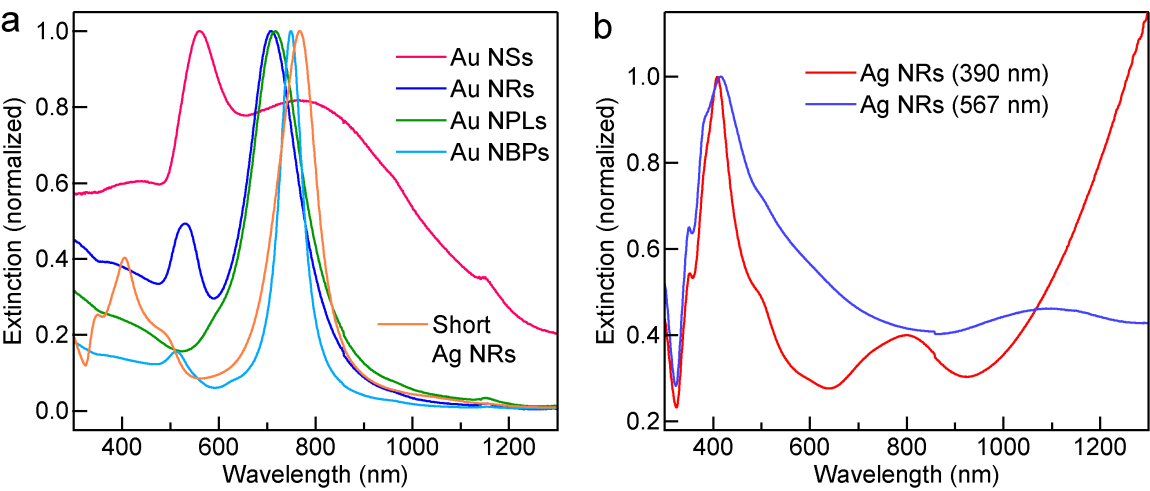


**Fig. S1 Extinction spectra.** **a** For the five samples shown in Fig. 1b. They are the Au nanospheres (Au NSs), Au nanorods (Au NRs), Au nanoplates (Au NPLs), Au nanobipyramids (Au NBPs) and short Ag nanorods (Ag NRs). Each spectrum has been normalized against its maximal extinction value. For the Au NS sample, the two plasmon peaks at 560 nm and 778 nm originate from the quadrupolar and dipolar plasmon modes, respectively. **b** For the two high-aspect-ratio Ag nanorod samples shown in Fig. 1c. Both spectra have been normalized against their maximal extinction values of the transverse plasmon mode at 400 nm, respectively. All of the nanoparticle samples were dispersed in aqueous solutions.


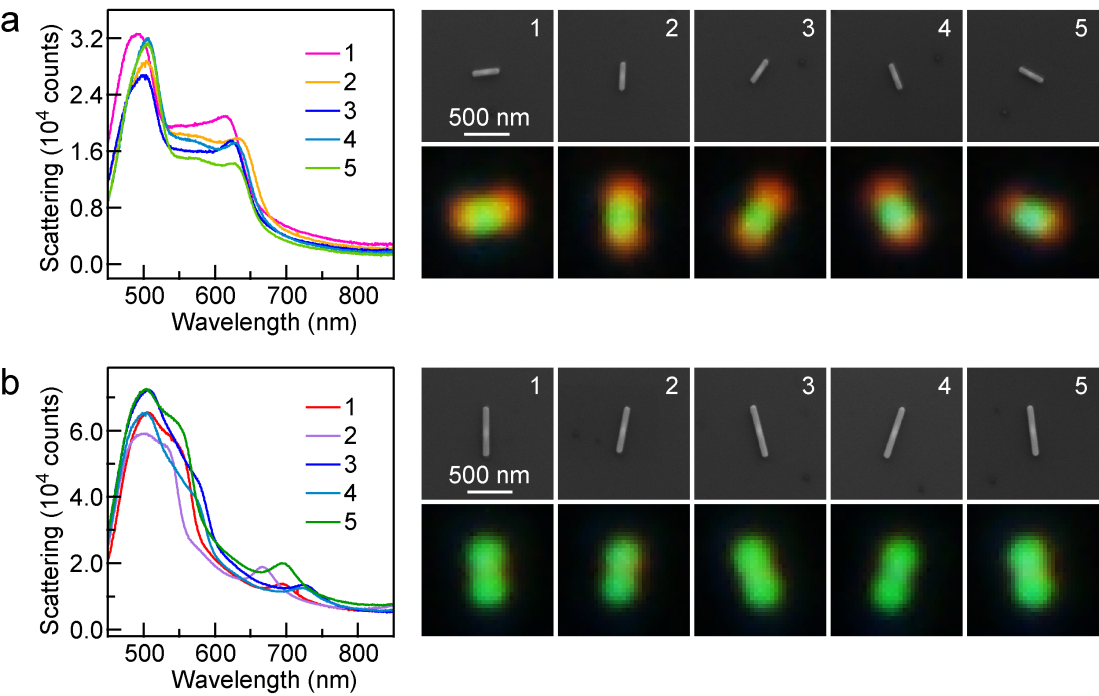


**Fig. S2 Verification of the orientations of the Ag nanorods.** **a** Dark-field scattering spectra (left), SEM (right upper row) and correlated dark-field (right bottom row) images measured from five representative Ag nanorods showing double red spots in different orientations. The used Ag nanorod sample has an average length/diameter of 303 ± 5 nm/64 ± 3 nm. **b** Dark-field scattering spectra (left), SEM (right upper row) and correlated dark-field (right bottom row) images measured from five representative Ag nanorods showing double green spots in different orientations. The used Ag nanorod sample has an average length/diameter of 559 ± 27 nm/66 ± 3 nm. The Ag nanorod samples were deposited on Si substrates with a 300-nm-thick thermal oxide layer.


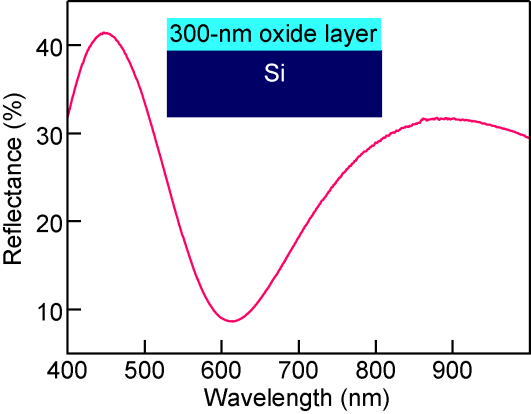


**Fig. S3 Reflectance spectrum of the oxide-coated Si wafer.** The inset shows the schematic of the substrate. The thickness of the thermal oxide layer is 300 nm. The spectrum shows two strong peaks at 450 nm and 900 nm, as well as a dip at 600 nm, all of which are caused by the thin film interference of the thermal oxide layer. As a result, when a scattering peak of the Ag nanorod is located close to the reflection peaks during the dark-field scattering measurement, the observed signal can be largely enhanced due to the reflection from the thermal oxide layer, especially in the short-wavelength region. Although the thermal oxide layer altered the dark-field scattering spectra and images in the short-wavelength region, the results obtained on the oxide-coated Si substrates confirmed the correlation between the orientations of the Ag nanorods and their dark-field images shown in Fig. 2.


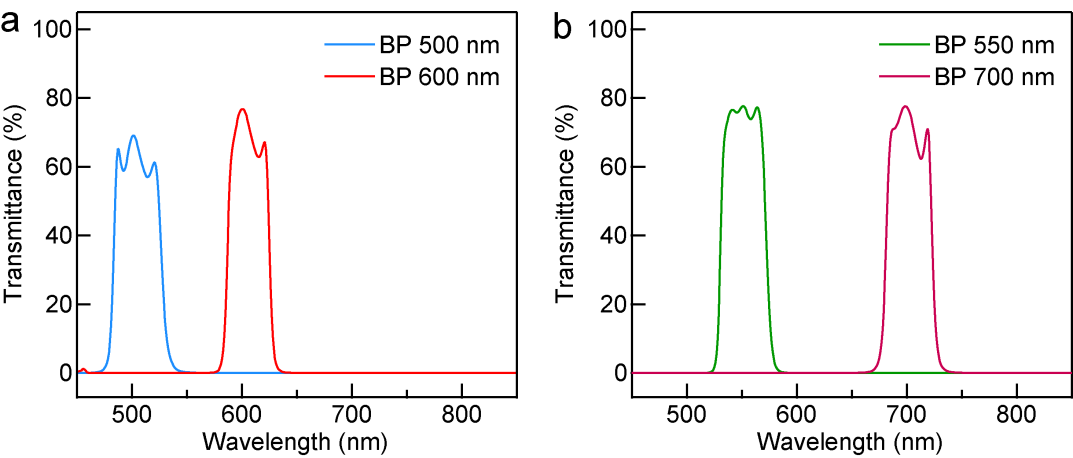


**Fig. S4 Transmittance spectra of the bandpass colour filters.** **a** For the 500-nm and 600-nm bandpass filters. **b** For the 550-nm and 700-nm bandpass filters. The full widths at half maximum of the transmittance peaks of the bandpass filters are all 40 nm.


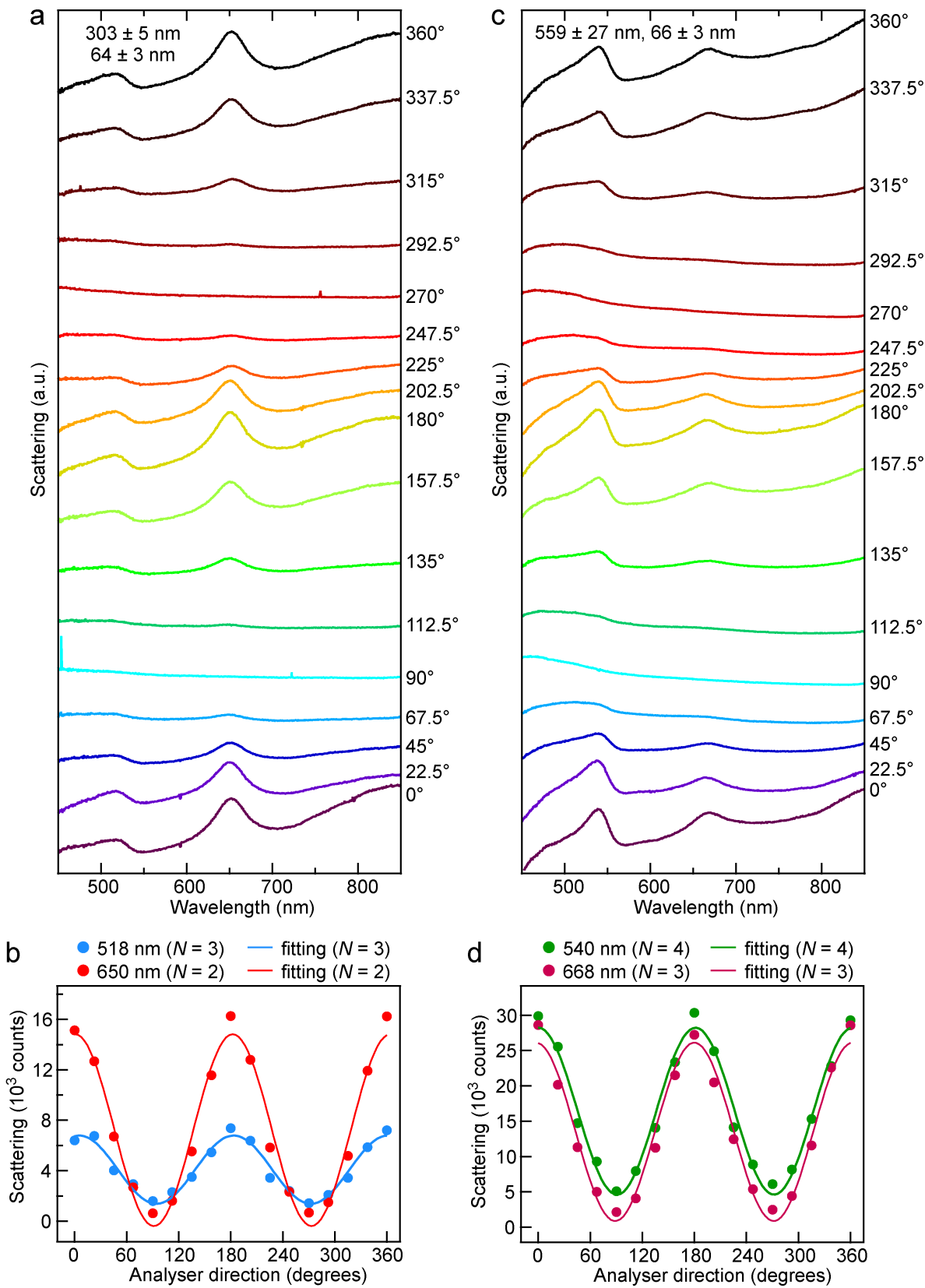


**Fig. S5 Emission polarization.** **a** Dark-field scattering spectra of a silver nanorod. The spectra were recorded as a function of the analyser polarization direction. The nanorod exhibits the octupolar and quadrupolar plasmon modes in the visible region. **b** Polarization dependences of the scattering intensities of the longitudinal octupolar (*N* = 3) and quadrupolar (*N* = 2) plasmon modes on the analyser polarization angle. The scattering intensities at 518 nm and 650 nm were extracted for the two modes, respectively. **c** Dark-field scattering spectra of a silver nanorod. The spectra were recorded as a function of the analyser polarization direction. The nanorod exhibits the hexadecapolar and octupolar plasmon modes in the visible region. **d** Polarization dependences of the scattering intensities of the longitudinal hexadecapolar (*N* = 4) and octupolar (*N* = 3) plasmon modes. The scattering intensities at 540 nm and 668 nm were extracted for the two modes, respectively. The Ag nanorods were supported on cover glass. The analyser polarization direction was varied from 0° to 360° at a step of 22.5°. The results in **a** and **b** are the raw data for the polar plots shown in Fig. 3a. The results in **c** and **d** are the raw data for the polar plots shown in Fig. 3b.


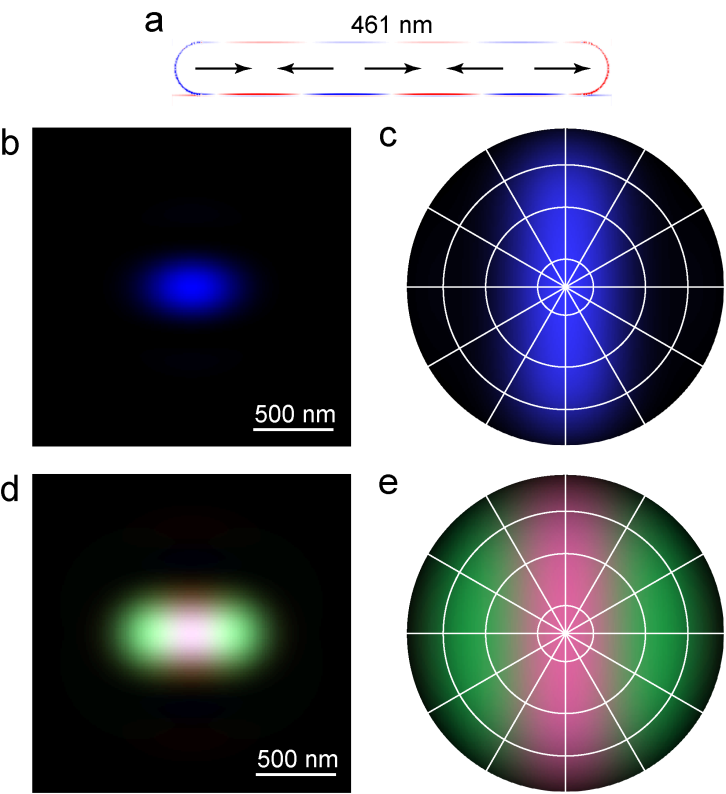


**Fig. S6 Simulation for the *N* = 5 plasmon peak.** **a** Simulated charge distribution contour. **b**, **c** Monochromic real-space and back-focal plane image for the *N* = 5 plasmon mode, respectively. **d**, **e** Overlapped real-space and back-focal plane image, respectively. The overlapped real-space and back-focal plane images were generated by combining the contributions from all of the three peaks shown in Fig. 4e. The peak wavelength is 461 nm. The Ag nanorod is shown in Fig. 4e.


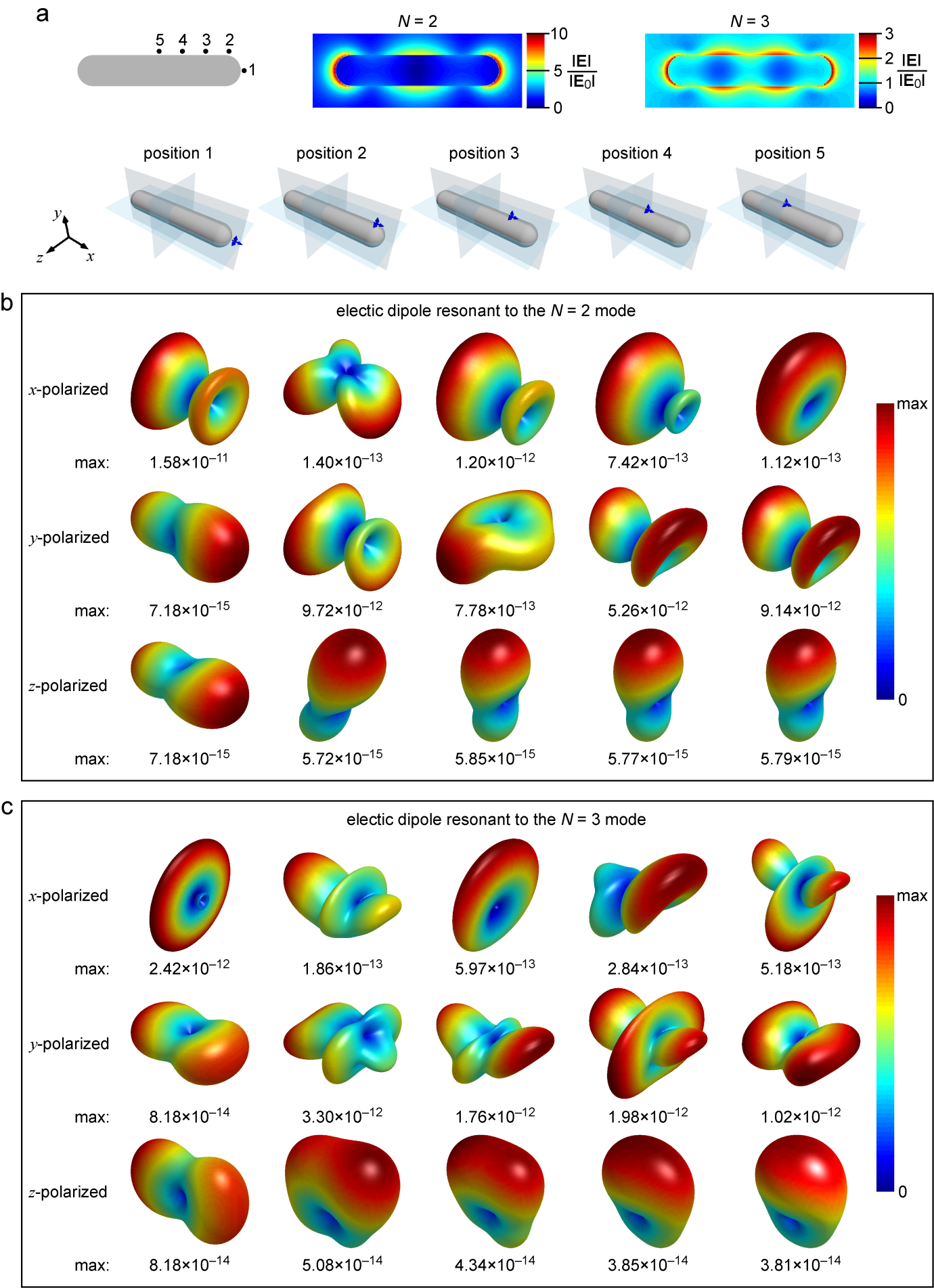


**Fig. S7 Radiation from an electric dipole emitter positioned on a silver nanorod.** **a** Schematics showing five representing positions of the emitter relative to the Ag nanorod, together with the localized electric field enhancement profiles of the quadrupolar (*N* = 2) and octupolar (*N* = 3) plasmon modes of the nanorod. The nanorod has a length of 350 nm and a diameter of 66 nm. The field enhancement contours are drawn at the linear scale. The electric field enhancement profiles were simulated under plane-wave excitation in order to display the different features of the two modes clearly. The position 1 is located at the end of the nanorod. The positions 2–5 are located on one side of the nanorod along the length axis with a spacing of 50 nm. They are at the central position in the direction perpendicular to the length axis. The dipole at all of the positions is 2 nm away from the surface of the Ag nanorod. Its orientation was set along the three axes of the displayed coordinate system, respectively, during the simulations. **b**, **c** FDTD-simulated 3D far-field radiation patterns of an electric dipole resonant to the *N* = 2 and *N* = 3 plasmon modes, respectively. The colour scales of the patterns are different, with the maximal value provided below each pattern. Among all of the cases, the *x*-oriented electric dipole emitter at the position 1 can efficiently couple to the *N* = 2 and *N* = 3 plasmon modes due to the strong local field enhancement at the two ends of the nanorod and the fact that the emitter has the same orientation as the electron oscillations of the longitudinal multipolar plasmon modes. In these cases, the radiation from the emitter to the far-field shows 3D far-field patterns similar to those shown in Fig. 6, suggesting that the dipolar emission can be efficiently modulated by the colour routing effect.
